# A Single Tertiary Center 14-year Experience With Mesenteric Panniculitis in Turkey: A Retrospective Study of 716 Patients

**DOI:** 10.5152/tjg.2023.22514

**Published:** 2023-02-01

**Authors:** Hüseyin Atacan, Murat Erkut, Ferhat Değirmenci, Selçuk Akkaya, Sami Fidan, Arif M. Coşar

**Affiliations:** 1 Department of Internal Medicine, Bulancak State Hospital, Giresun, Turkey; 2 Department of Gastroenterology, Karadeniz Technical University Faculty of Medicine, Trabzon, Turkey; 3 Department of Radiology, Health Sciences University, Trabzon Kanuni Training and Research Hospital, Trabzon, Turkey; 4 Department of Radiology, Karadeniz Technical University Faculty of Medicine, Trabzon, Turkey

**Keywords:** Computed tomography, malignancy, mesenteric panniculitis

## Abstract

**Background:**

Mesenteric panniculitis is a chronic inflammatory process seen in mesenteric tissue. The purpose of this study was to assess the prevalence, clinical, laboratory, and radiological findings, and malignancy in patients diagnosed with mesenteric panniculitis using computed tomography.

**Methods:**

A total of 716 patients with mesenteric panniculitis were retrospectively evaluated by screening all computed tomography scans performed between January 2005 and December 2018.

**Results:**

Among 65 278 patients undergoing CT, 716 were diagnosed with mesenteric panniculitis. The prevalence of mesenteric panniculitis was 1.1%. The mean age was 56 ± 14 (20-91) years. The malignant and nonmalignant groups comprised 354 (49.4%) and 362 (50.6%) patients, respectively. The mean age of the malignant group was significantly higher than the nonmalignant group (*P* < .001). The most common malignancy was breast cancer (12.2%). A history of abdominal surgery was present in 179 (25%) patients with mesenteric panniculitis and it is higher in the malignant group than the nonmalignant group (128 [36.1%], 51 [14%], respectively, *P* < .001). Mean hemoglobin level and leukocyte count were lower in the malignant group than in the nonmalignant group (*P* < .001, *P* < .001, respectively). The mean erythrocyte sedimentation rate was higher in the malignant group than in the nonmalignant group (*P* = .030). Radiological criterion 2 was less common and radiological criterion 5 was more common in the malignant group than the nonmalignant group (91.0%, 96.4%, *P* = .004; 35.9%, 27.1%, respectively, *P* = .011).

**Conclusions:**

It is recommended to conduct research for malignancy in patients with mesenteric panniculitis, especially in the presence of clinical, laboratory, and radiological findings with high-risk features.

**Main Points**

- The prevalence of mesenteric panniculitis (MP) was 1.1% based on our data.
- There is a particular association between MP and malignancy, especially with breast cancer, gynecological cancer, and colorectal cancer.
- The presence of pseudocapsule at computed tomography is observed more frequently in patients with malignancy.

## Introduction

Mesenteric panniculitis (MP) is a chronic inflammatory process frequently seen in the mesenteric tissue, and more rarely in the omentum and mesocolon.^1^ The incidence of MP has increased in recent years due to the more frequent clinical use of computed tomography (CT). Various studies have shown a prevalence of MP between 0.16% and 7.8% in patients who performed abdominal CT.^2-6^ Although the pathogenesis is uncertain, it is thought to be associated with mesenteric ischemia and autoimmune response.^7^ High erythrocyte sedimentation rate (ESR) and C-reactive protein (CRP) levels, leucocyte count, and low hemoglobin (hb) level may be observed in laboratory findings.^8^ Characteristic CT findings for MP include fatty mass lesion in the small intestine mesenteric tissue, the mesenteric adipose tissue with higher density than the surrounding abdominal tissue, lymph nodes in the mesenteric adipose tissue, halo sign around lymph node and vessels, and the presence of hyperdense pseudocapsule around the mesenteric adipose tissue. Fat necrosis, chronic inflammation, and fibrosis are observed on histopathological examination.^9,10^

Although the etiology of MP is not fully understood, it is thought to be associated with abdominal surgery, abdominal trauma, ischemia, infection, autoimmune disease, and malignancy.^1^ Previous studies have reported malignancy rates between 1% and 75% in patients with MP.^2,4,11-13^ The most common malignancies are lymphoma, melanoma, colon cancer, and prostate cancer.^1^ Whether this is a true paraneoplastic phenomenon is still uncertain. Some authors interpret that the increase in MP is an epiphenomenon due to the more frequent use of CT in patients with cancer.^1,7^

Until now, there are many studies evaluating the clinical and radiological findings and underlying disease in patients with MP. However, larger studies are needed due to the limited number of patients. The purpose of this study was to assess the prevalence, clinical, laboratory, and radiological findings, underlying chronic disease, and in particular, the association with malignancy in patients diagnosed with MP using CT.

## Materials and Methods

### Patients

**Figure 1.**
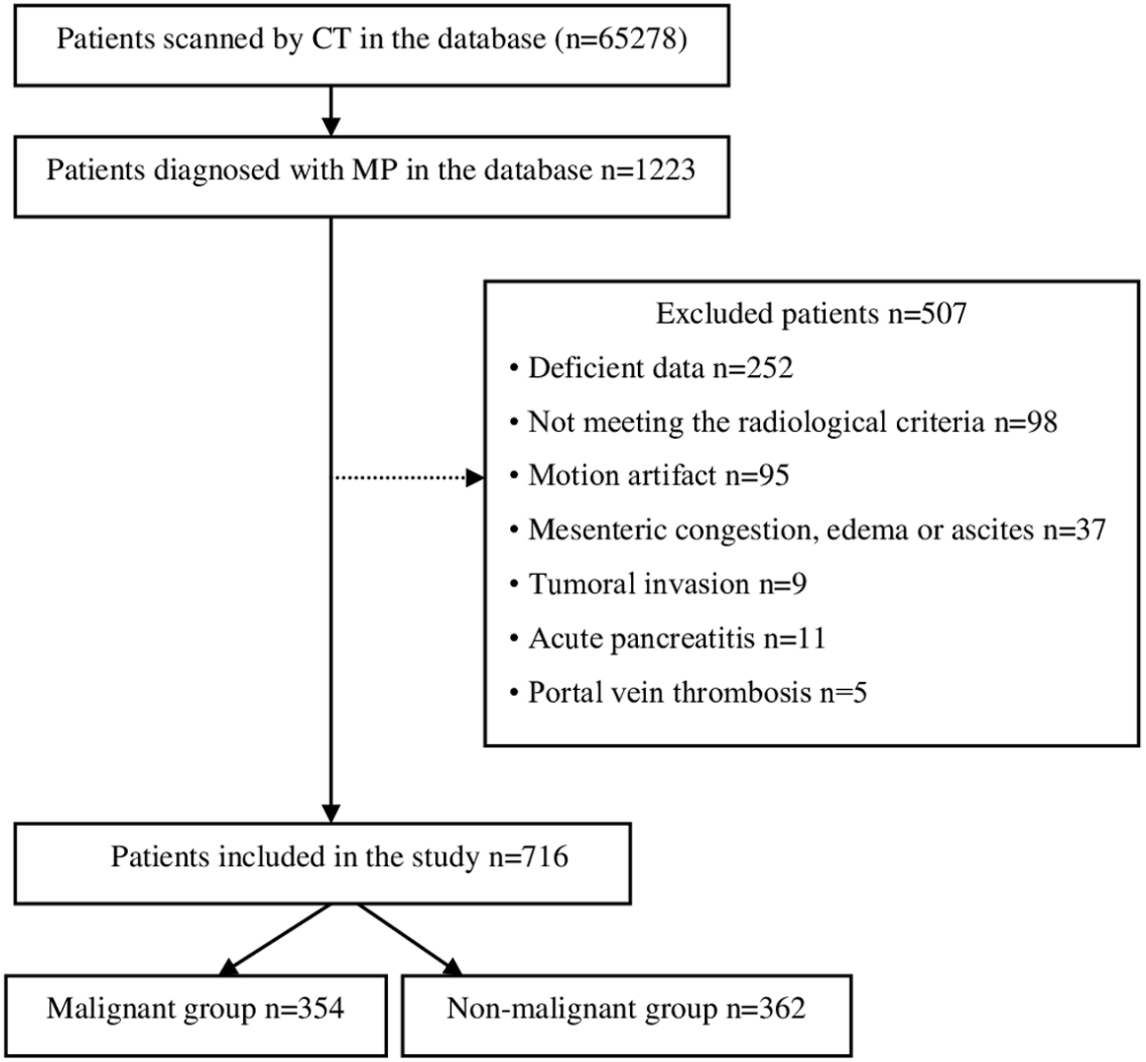
Consort diagram.

The data of 1223 patients who have reported diagnosis of MP in 65 278 abdominal CT scans performed for any reason at the Karadeniz Technical University between January 2005 and December 2018 were retrospectively evaluated using the hospital database. Around 507 patients were excluded from the study for reasons such as missing demographic data, suboptimal evaluation due to motion artifact, failure to meet the diagnosis of MP in re-evaluation by the radiologist based on reference criteria, the presence of mesenteric congestion or edema, ascites, tumoral invasion, portal vein thrombosis, and acute pancreatitis (Figure 1). Approval for the study was granted by Karadeniz Technical University Medical Faculty Ethical Committee of Scientific Research on March 7, 2019.

A total of 716 patients were included in the study. The patients were divided into 2 groups as malignant and nonmalignant, according to the presence of concomitant malignancy or not. Patients’ demographic data, underlying chronic disease, history of abdominal surgery, laboratory tests (complete blood count, electrolytes, renal function tests, liver function tests, lactate dehydrogenase, uric acid, albumin, amylase, acute phase reactants, ferritin, cholesterol, international normalized ratio), and radiological findings were recorded. Laboratory tests performed simultaneously with radiological diagnosis or within 12 weeks were evaluated.

### Radiological Evaluation

Computed tomography images were obtained using a 160-section scanner (Toshiba Aquilion, Toshiba Medical Systems, Japan) and a 16-section scanner (SOMATOM Sensation 16, Siemens, Forchheim, Germany). Standard oral or intravenous contrast material examination protocol was applied to all patients according to the indications. Contrast material was not applied in cases of suspicion of renal stone, contrast allergy, or renal failure. Following the administration of 100-120 mL non-ionic contrast material and 30 mL saline injection at 4 mL/h, images were obtained in the portal venous phase after a 70-second waiting period. The CT protocol involved a voltage of 120 kW, 150-165 mass tube flow, 2.5 mm collimation, a section thickness of 2 mm, and a rotation duration of 0.5 seconds.

Evaluation of CT images was made by 2 radiologists blinded to the patients’ diagnoses. The number of positive criteria and which criteria were positive in each patient were recorded separately. The presence of 3 out of 5 findings according to the Coulier classification was considered sufficient for the diagnosis of MP.^14,15^ These criteria were as follows: a well-defined mesenteric fatty mass lesion (criterion 1), the presence of mesenteric adipose tissue with higher density than the surrounding abdominal tissue (criterion 2), the presence of blood vessels and small lymph nodes (criterion 3), halo sign (fat ring) (criterion 4), and pseudocapsule (criterion 5).

### Statistical Analysis

Data analysis was performed using Statistical Package of Social Sciences version 23.0 software (IBM Corp.; Armonk, NY, USA). The results were expressed as numbers and percentages for categorical variables, and mean, standard deviation, minimum, and maximum values for measurement variables. The normality of the distribution of variables was evaluated using the Kolmogorov–Smirnov test. The chi-square test was used for categorical and ordinal variables. The Mann–Whitney *U*-test was employed for nonparametric variables. Student’s *t*-test was used for parametric variables. *P* < .05 was considered as statistically significant.

## Results

### Patients

Around 716 of 65 278 patients undergoing CT imaging for any reason were diagnosed with MP. The prevalence of MP was 1.1%.

The mean age was 56 ± 14 (20-91) years. Out of 716 patients, 408 (57%) were women and 308 (43%) were men. There were 354 (49.4%) patients in the malignant group and 362 (50.6%) patients in the nonmalignant group. The mean age was higher in the malignant group than in the nonmalignant group (59 ± 11 years and 53 ± 16 years, respectively, *P* < .001). Peak age range was 60-69 years (32.5%) in the malignant group and 50-59 years (26.5%) in the nonmalignant group. There were 213 (60%) women and 141 (40%) men in the malignant group, and 195 (54%) women and 167 (46%) men in the nonmalignant group. There was no statistically significant difference between the 2 groups in terms of gender (*P* = .089) (Table 1).

### Mesenteric Panniculitis and Malignant Diseases

Most common malignancies detected in patients with MP were breast cancer (12.2%), gynecological cancer (6.4%), and colorectal cancer (5.6%). In addition, 24.4% of malignancies were located in the intra-abdominal region (Table 2).

### Mesenteric Panniculitis and Nonmalignant Diseases

Around 210 (29.3%) patients had a history of hypertension (HT), 107 (14.9%) had diabetes mellitus (DM), and 77 (10.8%) had coronary artery disease in MP patients. Rheumatologic disease was observed less frequently with 18 (2.5%) patients. Nonmalignant diseases accompanying MP are shown in Table 2.

### Mesenteric Panniculitis and Surgery

There was a history of abdominal surgery in 179 (25%) patients with MP, 128 (36.1%) in the malignant group, and 51 (14%) in the nonmalignant group. Abdominal surgery history was statistically higher in the malignant group than in the nonmalignant group (*P* < .001). On the other hand, if the patients with intra-abdominal malignancy are not inoperable or unresectable, the need for tumor surgery is inevitable. Considering this situation, a history of intra-abdominal surgery which was not due to tumor itself was detected in 29 (8.1%) patients in the malignant group. The most common type of surgery among patients with MP was gynecological at a rate of 8.4%. Frequencies according to types of intra-abdominal surgery are shown in Table 3.

### Laboratory Findings

Mean hb level and leukocyte count were lower in the malignant group than in the nonmalignant group (hb; 12.7 ± 1.6 g/dL and 13.3 ± 1.8 g/dL, *P* < .001, leukocyte; 7.2 ± 6.6 × 10^3^/µL and 8.1 ± 3.1 × 10^3^/µL, respectively, *P* < .001). The frequency of anemia in patients with MP was 29.5%, and it was higher in the malignant group than in the nonmalignant group (33.9% and 25.2%, respectively; *P* = .011). Additionally, the frequency of leukopenia in patients with MP was 14.7%, and it was higher in the malignant group than the nonmalignant group (19.8% and 9.7%, respectively; *P* < .001). Mean ESR was higher in the malignant group than the nonmalignant group (27.5 ± 22.8 mm/h and 21.8 ± 19.2 mm/h, respectively, *P* = .030). The frequency of high ESR in patients with MP was 48.3%, and it was also higher in the malignant group than in the nonmalignant group (53.1% and 42.2%, respectively; *P* = .017). Mean calcium level was higher in the malignant group than in the nonmalignant group (9.4 ± 0.6 mg/dL and 9.2 ± 0.6 mg/dL, respectively, *P* = .004). Mean alanine aminotransferase (ALT) and aspartate aminotransferase (AST) were lower in the malignant group than in the nonmalignant group (ALT; 25.5 ± 43.6 U/L and 28.3 ± 36.6 U/L, *P* = .023, AST; 27.2 ± 19.5 U/L and 29.9 ± 23.4 U/L, respectively, *P* = .016) (Table 4).

### Radiological Findings

The presence of mesenteric adipose tissue with higher density than the surrounding abdominal tissue (criterion 2) was lower in the malignant group than in the nonmalignant group (91.0% and 96.4%, respectively, *P* = .004). The presence of a pseudocapsule (criterion 5) was higher in the malignant group than in the nonmalignant group (35.9% and 27.1%, respectively, *P* = .011). The presence of blood vessels and small lymph nodes (criterion 3) was determined in all patients’ CT findings and no statistically significant difference was observed between the 2 groups in terms of findings of a well-defined mesenteric fatty mass lesion (criterion 1) and halo sign (fat ring) (criterion 4) (Table 5).

The frequency of detection of any 3, 4, or all 5 radiological criteria in all patients was 57.9% (n = 415), 32.1% (n = 230), and 9.9% (n = 71), respectively. This rate was 58.2% (n = 206), 31.4% (n = 111), 10.5% (n = 37) in the malignant group, and 57.7% (n = 209), 32.9% (n = 119), 9.4% (n = 34) in the nonmalignant group, respectively. No significant difference was observed between the two groups (*P* = .845) (Table 6).

## Discussion

The prevalence of MP was found to be 0.16% by Wilkes et al,^3^ 0.58% by Gögebakan et al,^5^ and 2.5% by van Putte-Katier et al.^15^ On the other hand, the highest prevalence in the literature was determined as 7.83% by Coulier et al.^6^ The prevalence of MP in our study was 1.1%, and this finding is compatible with the literature in general.

In a systemic review, the mean age at the diagnosis of MP was 62 years, with 70% male and 30% female patients.^16^ The mean age at the diagnosis in our study was 56 years, which was similar to the literature. We found that the female gender was predominant in patients with MP (1.3/1). In agreement with our study, Daskalogiannaki et al^2^ reported a female/male ratio of 1.9/1.

There are conflicting results between MP and cancer. Cancer was determined in 38% of patients with MP in 1 systematic review.^16^ van Putte-Katier et al^15^ reported that the possibility of malignancy in patients with MP and the development of malignancy in the 5-year follow-up period is higher than in the control group. In some studies, it is thought that MP is a paraneoplastic syndrome that develops as a result of an inappropriate response to the inflammation observed in malignancy.^3^ However, Gögebakan et al^5^ and Buchwald et al^7^ reported that there was no relationship between MP and cancer, and it was an epiphenomenon rather than a true paraneoplastic event. The most common types of cancer in patients with MP in various studies are lymphoma, melanoma, colon cancer, and prostate cancer.^1^ Malignancy was present in 49% of patients with MP in our study. Moreover, the most common types of cancer were breast, colorectal, and gynecological cancer in patients with MP. Compared with other studies, breast cancer was observed more common and lymphoma was less common. Similar to the present study, breast cancer was also found the most common malignancy by Al-Omari et al.^10^ Therefore, we believe that the possibility of breast cancer should also be taken into account in cases of MP in women. In addition, the higher incidence of breast cancer in our study explains the higher rate of MP in the female gender.

Studies have shown older age in MP with accompanying malignancy compared to those without. In the study of Al-Omari et al^10^ peak age range of MP in patients with accompanying malignancy was between 60 and 69 years. Similarly, in our study, MP was observed at an older age in the malignant group than in the nonmalignant group, and the peak age range of MP in the malignant group was 60-69.

The prevalence of HT and DM in Turkey was 31.8% and 13.7%, respectively.^17,18^ Canyiğit et al^19^ reported that the prevalence of HT and DM was 35.2% and 21.5%, respectively, in patients with MP. Similarly, Gögebakan et al^5^ determined them to be 40.3% and 26.0%, respectively.^5^ In our study, 33.2% of patients with MP had HT and 16.9% had DM. These data were compatible with the literature.

The rate of abdominal surgery in patients with MP was determined to be 46% by van Putte-Katier et al ^15^ and 45% by Wilkes et al.^3^ Both studies hypothesized that the abnormal healing response after tissue damage due to surgery may cause MP. In our study, the history of abdominal surgery was present in 25% of patients, lower than in other studies in the literature. The most common types of surgery in patients with MP are cholecystectomy, appendectomy, hysterectomy, and colectomy.^1^ Similarly, we found that the most common types of surgery were gynecological surgery, colectomy, and cholecystectomy. Additionally, surgery was performed more frequently in the malignant group than in the nonmalignant group in our study. However, when patients who had undergone tumor surgery were excluded, the frequency of intra-abdominal surgery in patients with the malignant group was similar to the rate of nonmalignant patients.

MP is a clinical problem characterized by chronic inflammation. Canyiğit et al^19^ and Sharma et al^20^ showed an increase in acute phase reactants, while Gögebakan et al^5^ and Badet et al^13^ determined no increase in acute phase reactants in patients with MP. In our study, we found mild elevation in acute phase reactants. In addition, mean leukocyte level was lower and mean ESR level was higher in the malignant group compared to the nonmalignant group. We thought that a low leukocyte level in the malignant group may be related to the underlying malignancy itself or bone marrow suppression secondary to chemotherapy and/or radiotherapy. Some studies have shown an increase in the frequency of anemia in patients with MP.^5,20^ Otherwise, Wilkes et al^3^ have found no relationship and van Putte-Katier et al^15^ observed normal hb level in patients with MP, but lower levels in malignant patients than nonmalignant patients. Similarly, in our study, mean hb level was within the normal range, and lower in the malignant group than in the nonmalignant group. In addition, the frequency of anemia was observed to be higher in the malignant group compared to the nonmalignant group. We think that the presence of anemia in the malignant group may be related to the tumor itself and/or chemotherapeutic agents used in the medical therapy in patients with MP.

In 2011, Coulier et al^6^ published radiological diagnostic criteria for the diagnosis of MP, which consists of 5 specific CT findings. Several studies have identified criteria 1, 2, and 3 in all patients with MP.^6,10,13^ Similarly, criteria 1 and 2 were observed in more than 90% of patients, and criterion 3 was determined in all patients at CT in our study. Criterion 4 has been defined as specific for the diagnosis of MP.^6^ Al-Omari et al^10^ reported that criterion 4 was detected in 86% of patients with MP.^10^ Badet et al^13^ detected criterion 4 and 5 in 56% and 59% of patients, respectively.^[Bibr b13-tjg-34-2-140]^ In our study, criterion 4 and 5 were observed at rates of 28% and 31%, respectively, these rates were less than other studies. In the study of Scheer et al.^21^ criterion 4 was detected in 58% of patients with malignancy, and 69.0% of patients without malignancy in MP. Similarly, Wilkes et al^3^ reported a relative increase in potential malignancy in the absence of criterion 4. In our study, there was no difference in terms of criterion 4 between the malignant and nonmalignant groups. Al-Omari et al^10^ reported that criterion 5 was detected more frequently in MP patients with malignancy compared to nonmalignant patients. On the contrary, Scheer et al^21^ found that criterion 5 was seen in 69.3% of MP patients, and this finding was more common in the nonmalignant group. In our study, criterion 5 was more frequent in the malignant group compared to the nonmalignant group. Therefore, when criterion 5 is detected, it is important to consider that there may be a potential for a concomitant malignancy in patients with MP.

The principal limitation of this study is that diagnosis of MP was based on radiological criteria alone, and making a histopathological diagnosis was not possible. Secondly, the study design was retrospective, and thirdly, the follow-up period was not standardized. Due to lack of optimal follow-up data, it can be said that the possibility of subsequent malignancy in the nonmalignant group could not be evaluated properly.

## Conclusions

The prevalence of MP was 1.1% based on our data. There is a particular association between MP and malignancy, especially with breast cancer, gynecological cancer, and colorectal cancer. The presence of pseudocapsule at CT is observed more frequently in patients with malignancy. 

Considering our data, clinicians and radiologists should be careful about malignancy in patients with MP, patients should be followed closely, especially in patients with clinical, laboratory, and radiological high-risk features even in the absence of a history of malignancy.

## Figures and Tables

**Table 1.** Demographic Characteristics in the Patients with MP

Characteristics	Total (n = 716)	Malignant Group (n = 354)	Nonmalignant Group (n = 362)	*P*
Age years, (mean ± SD)	56 ± 14	59 ± 11	53 ± 16	<.001*
Gender n, (%)				.089
Female	408 (57)	213 (60)	195 (54)	
Male	308 (43)	141 (40)	167 (46)	

MP, mesenteric panniculitis; SD, standard deviation.

*Statistically significant.

**Table 2.** Malignant and Nonmalignant Diseases in Patients with Mesenteric Panniculitis

Type of cancer	n (%)
Breast cancer	87 (12.2)
Gynecological cancer	46 (6.4)
Colorectal cancer	40 (5.6)
Lymphoma	37 (5.2)
Gastric cancer	29 (4.1)
Lung cancer	19 (2.7)
Renal cell cancer	19 (2.7)
Prostate cancer	18 (2.5)
Pancreatic cancer	8 (1.1)
Chronic lymphoid leukemia	8 (1.1)
Bladder cancer	7 (1)
Acute leukemia	6 (0.8)
Other*	30 (4.2)
**Type of disease**	**n (%)**
Hypertension	210 (29.3)
Diabetes mellitus	107 (14.9)
Coronary artery disease	77 (10.8)
Cerebrovascular disease	18 (2.5)
Bronchial asthma	12 (1.7)
Peripheral artery disease	12 (1.7)
Cirrhosis	8 (1.1)
Rheumatoid arthritis	7 (1)
Inflammatory bowel disease	3 (0.4)
Spondyloarthropathy	3 (0.4)
Systemic lupus erythematosus	2 (0.3)
Ankylosing spondylitis	2 (0.3)
Behçet’s disease	2 (0.3)
Mycosis fungoides	2 (0.3)
Sarcoidosis	1 (0.1)
Multiple sclerosis	1 (0.1)
Hypersensitivity angiitis	1 (0.1)
Gout	1 (0.1)
Graves’ disease	1 (0.1)
Psoriatic arthritis	1 (0.1)
Diverticulitis	1 (0.1)

*Malignant melanoma, multiple myeloma, basal cell cancer, cholangiocellular cancer, parotid gland cancer, nasopharyngeal cancer, thyroid cancer, testis cancer, soft tissue sarcoma, germ cell tumor, neuroendocrine tumor, laryngeal cancer, glioblastoma multiforme.

**Table 3.** Abdominal Surgeries in Patients with Mesenteric Panniculitis

Type of surgery	n (%)
Gynecological surgery	60 (8.4)
Colectomy	32 (4.5)
Cholecystectomy	28 (3.9)
Gastrectomy	19 (2.6)
Nephrectomy	15 (2.1)
Appendectomy	12 (1.7)
Hernioplasty	3 (0.4)
Whipple surgery	3 (0.4)
Liver resection	2 (0.3)
Splenectomy	2 (0.3)
Cystectomy	2 (0.3)
Cesarean	1 (0.1)

**Table 4.** Laboratory Results in Patients with MP

Values	Total	Malignant Group	Nonmalignant Group	*P*
Hb g/dL, (mean ± SD)	13.0 ± 1.7	12.7 ± 1.6	13.3 ± 1.8	<.001*
Leukocyte × 10^3^/µL (mean ± SD)	7.6 ± 5.2	7.2 ± 6.6	8.1 ± 3.1	<.001*
Platelet × 10^3^/µL (mean ± SD)	240.7 ± 80.2	236.9 ± 81.9	244.4 ± 78.5	.180
Creatinine, mg/dL (mean ± SD)	0.8 ± 0.3	0.8 ± 0.3	0.8 ± 0.3	.497
Uric acid, mg/dL, (mean ± SD)	5.3 ± 1.6	5.2 ± 1.5	5.3 ± 1.6	.636
ALT, U/L (mean ± SD)	26.9 ± 40.4	25.5 ± 43.6	28.3 ± 36.6	.023*
AST, U/L (mean ± SD)	28.5 ± 21.5	27.2 ± 19.5	29.9 ± 23.4	.016*
Calcium, mg/dL (mean ± SD)	9.3 ± 0.6	9.4 ± 0.6	9.2 ± 0.6	.004*
Amylase, U/L (mean ± SD)	68.7 ± 39.7	69.5 ± 42.9	67.7 ± 35.3	.539
Bilirubin, mg/dL (mean ± SD)	0.8 ± 1.4	0.8 ± 1.6	0.8 ± 0.9	.980
GGT, U/L (mean ± SD)	51.8 ± 126.4	53.1 ± 148.7	50.1 ± 90.3	.850
LDH, U/L (mean ± SD)	225.0 ± 105.9	221.3 ± 110.7	230.6 ± 98.3	.171
INR (mean ± SD)	1 ± 0.2	1 ± 0.2	1 ± 0.1	.880
Albumin, g/L, (mean ± SD)	4.0 ± 0.5	4.1 ± 0.5	4.0 ± 0.5	.977
ESR, mm/h (mean ± SD)	25.0 ± 21.5	27.5 ± 22.8	21.8 ± 19.2	.030*
CRP, mg/L (mean ± SD)	2.0 ± 3.7	1.9 ± 3.8	2 ± 3.7	.622
Ferritin, µg/L (mean ± SD)	144.3 ± 252.8	165.2 ± 290.4	123.2 ± 207.9	.132
**Frequency**				
Anemia, n (%)	211 (29.5)	120 (33.9)	91 (25.2)	.011*
Leukopenia, n (%)	105 (14.7)	70 (19.8)	35 (9.7)	<.001*
High ALT, n (%)	77 (11.6)	32 (9.4)	45 (13.8)	.081
High AST, n (%)	117 (17.8)	51 (15.1)	66 (20.6)	.063
High calcium, n (%)	7 (1.3)	6 (1.9)	1 (0.4)	.086
High ESR, n (%)	234 (48.3)	145 (53.1)	89 (42.2)	.017*

ALT, alanine aminotransferase; AST, aspartate aminotransferase; CRP, C-reactive protein; ESR, erythrocyte sedimentation rate; GGT, gamma glutamyl transferase; Hb, hemoglobin; INR, international normalized ratio; LDH, lactate dehydrogenase; MP, mesenteric panniculitis; SD, standard deviation.

*Statistically significant.

**Table 5.** Radiological Diagnostic Criteria in Patients with Mesenteric Panniculitis

Criteria	Total (%)	Malignant n (%)	Nonmalignant n (%)	*P*
1	706 (98.6)	349 (98.6)	357 (98.6)	1.000
2	671 (93.7)	322 (91.0)	349 (96.4)	.004*
3	716 (100)	354 (100)	362 (100)	1.000
4	202 (28.2)	95 (26.8)	107 (29.6)	.418
5	225 (31.4)	127 (35.9)	98 (27.1)	.011*

Criterion 1, a well-defined mesenteric fatty mass lesion; criterion 2, the presence of mesenteric adipose tissue with higher density than the surrounding abdominal tissue; criterion 3, the presence of blood vessels and small lymph nodes; criterion 4, halo sign (fat ring); criterion 5, the presence of a pseudocapsule.

*Statistically significant.

**Table 6.** The Number of Radiological Criteria in Patients with Mesenteric Panniculitis

Criterion number	Total n (%)	Malignant Group n (%)	Non-malignant Group n (%)	*P*
3	415 (57.9)	206 (58.2)	209 (57.7)	
4	230 (32.1)	111 (31.4)	119 (32.9)	.845
5	71 (9.9)	37 (10.5)	34 (9.4)	

Criterion 3, the presence of blood vessels and small lymph nodes; criterion 4, halo sign (fat ring); criterion 5, the presence of a pseudocapsule.
